# Laboratory Test to Evaluate the Resistance of Cementitious Materials to Biodeterioration in Sewer Network Conditions

**DOI:** 10.3390/ma14030686

**Published:** 2021-02-02

**Authors:** Amr Aboulela, Matthieu Peyre Lavigne, Amaury Buvignier, Marlène Fourré, Maud Schiettekatte, Tony Pons, Cédric Patapy, Orlane Robin, Mansour Bounouba, Etienne Paul, Alexandra Bertron

**Affiliations:** 1 LMDC, Université de Toulouse, UPS, INSA, 31077 Toulouse, France; aboulela@insa-toulouse.fr (A.A.); amaury.buvignier@gmail.com (A.B.); fourre@insa-toulouse.fr (M.F.); schiette@insa-toulouse.fr (M.S.); tony.pons@outlook.fr (T.P.); patapy@insa-toulouse.fr (C.P.); orlane.robin@live.fr (O.R.); 2 TBI, Université de Toulouse, CNRS, INRAE, INSA, 31077 Toulouse, France; mpeyrela@insa-toulouse.fr (M.P.L.); bounouba@insa-toulouse.fr (M.B.); paul@insa-toulouse.fr (E.P.)

**Keywords:** mortar, biodegradation, sewer, sulfuric acid, microorganisms, cementitious materials, durability

## Abstract

The biodeterioration of cementitious materials in sewer networks has become a major economic, ecological, and public health issue. Establishing a suitable standardized test is essential if sustainable construction materials are to be developed and qualified for sewerage environments. Since purely chemical tests are proven to not be representative of the actual deterioration phenomena in real sewer conditions, a biological test–named the Biogenic Acid Concrete (BAC) test–was developed at the University of Toulouse to reproduce the biological reactions involved in the process of concrete biodeterioration in sewers. The test consists in trickling a solution containing a safe reduced sulfur source onto the surface of cementitious substrates previously covered with a high diversity microbial consortium. In these conditions, a sulfur-oxidizing metabolism naturally develops in the biofilm and leads to the production of biogenic sulfuric acid on the surface of the material. The representativeness of the test in terms of deterioration mechanisms has been validated in previous studies. A wide range of cementitious materials have been exposed to the biodeterioration test during half a decade. On the basis of this large database and the expertise gained, the purpose of this paper is (i) to propose a simple and robust performance criterion for the test (standardized leached calcium as a function of sulfate produced by the biofilm), and (ii) to demonstrate the repeatability, reproducibility, and discriminability of the test method. In only a 3-month period, the test was able to highlight the differences in the performances of common cement-based materials (CEM I, CEM III, and CEM V) and special calcium aluminate cement (CAC) binders with different nature of aggregates (natural silica and synthetic calcium aluminate). The proposed performance indicator (relative standardized leached calcium) allowed the materials to be classified according to their resistance to biogenic acid attack in sewer conditions. The repeatability of the test was confirmed using three different specimens of the same material within the same experiment and the reproducibility of the results was demonstrated by standardizing the results using a reference material from 5 different test campaigns. Furthermore, developing post-testing processing and calculation methods constituted a first step toward a standardized test protocol.

## 1. Introduction

The need to develop efficient systems for the transportation and treatment of sewage worldwide with sustainable infrastructure has become a significant challenge for modern society. One major obstacle is the reduced durability of cementitious materials due to biodeterioration phenomena.

The biodeterioration of concrete pipelines in sewer environments is considered as a major health, environmental, and economic issue. On a European level, the European directive (Directive 91/271/CEE) imposes that agglomerations with >2000 habitants be equipped with wastewater treatment plants. On an international level, in 2008, the United Nations fixed the aim of reducing the number of people without safe water and clean sanitation by half. The World Health Organization (WHO) reported that approximately 2 billion people were still lacking basic sanitation services in 2017 [1].

Sewer infrastructure is made mainly of cement-based materials, which suffer from deterioration during their service-life [2]. Ten percent of the deterioration of concrete structures in sewage environments has been linked to biodeterioration induced by biochemical processes [2]. The release of hydrogen sulfide (H_2_S) and its biological oxidation into sulfuric acid (H_2_SO_4_) are considered to be at the origin of the deterioration of cementitious materials in sewer environments. 

H_2_S production in sewage comes from the activity of sulfate-reducing bacteria (SRB) growing in anaerobic conditions [3,4,5,6,7]. H_2_S is released into the gas area of the sewer pipe and reacts with the concrete surface (along with CO_3_^2−^), causing the surface pH to fall below 9 [6]. In contact with the concrete, sulfur compounds from H_2_S are oxidized into sulfuric acid by neutrophilic and then by acidophilic sulfur-oxidizing bacteria (SOB), which decreases the surface pH to approximately 2 [3,6,8,9,10,11]. The deterioration of the cementitious matrix by sulfuric acid is a coupled attack by the acid H^+^ and the sulfate ions SO_4_^2−^. The reaction of the acid with the cementitious matrix leads to the decalcification and the dissolution of calcium-bearing phases–mainly portlandite (CH) and C-A-S-H for Portland cement (PC)-based materials and katoite (C_3_AH_6_) for calcium aluminate cement (CAC)-based materials–and the residual anhydrous phases [12,13,14]. Such decalcification results in the formation of an amorphous silica gel for PC-based materials and alumina gel (AH_3_) for CAC-based materials. The penetration of sulfate inside the cement pores leads to the precipitation of expansive secondary sulfate-based phases. While the main identified precipitated sulfate salt is gypsum [15,16,17], secondary ettringite has been reported in several studies, mainly at the interface between the highly deteriorated concrete layer and the sound concrete, where strong alkaline conditions are maintained with the presence of large amounts of sulfate in the pore solution [16,17].

The current chemical tests have been demonstrated not to be representative of the actual deterioration phenomena encountered in sewer networks. For instance, the ranking of the performance of cementitious materials using purely chemical tests was different from the results obtained with in situ conditions [18,19]. Moreover, most chemical tests consist of immersing the specimens in a sulfuric acid solution with a regulated pH. However, such methods have shown that the aggressive solution becomes saturated in calcium sulfate very quickly, which slows down the attack and results in a decrease of acid consumption rate and less mass loss [19,20]. Moreover, the saturation conditions of the solution are unfavorable for the precipitation of sulfate-based salts that are damaging to the cementitious matrix. Thus, non-immersion conditions also contribute physically to the intensity of the deterioration phenomena.

The differences identified between the purely chemical attack and the biological attack can be attributed to a specific effect of the biofilm. Several studies have shown that the solid cementitious material has an influence on the development of the biofilm on its surface. For instance, the dissolution of different hydrated phases leads to the release of hydroxide ions, which neutralize the acid. Such a phenomenon could modify the pH of the medium and, so, disturb the development of bacteria on the concrete surface [13,19,21].

Moreover, the deteriorated concrete layer could have an impact on the deterioration process. In the case of biological attack, the deteriorated zone could create a suitable environment for the growth of bacteria due to its high porosity and the low pH of the zone. Such conditions might lead to the penetration of the bacteria into the layer and the production of more sulfuric acid near the sound concrete [20,22]. In the case of chemical attack by immersion, the porosity of the deteriorated zone is partly clogged by the precipitation of secondary phases, mainly gypsum [20,23]. Lately, it has been suggested that *Acidithiobacillus ferrooxidans* could produce more sulfuric acid in deeper anaerobic layers of the biofilm. However, the decisive role of such bacteria and their impact on the deterioration processes are not yet well understood [22]. In conclusion, chemical tests do not reproduce the attack conditions imposed by the presence of bacteria which contribute to the explanation of the differences in the results found with in situ conditions.

Two types of biological tests were actively developed in the literature. The test from the University of Hamburg–and similar tests from Gustave Eiffel University (France) and the Fraunhofer Society (Germany)–consist of exposing the materials for at least 6 months in an H_2_S atmosphere under controlled H_2_S concentration, temperature, and humidity conditions by regularly spraying an artificial consortium of bacteria, identified historically in Hamburg’s sewer network, onto the surface of the cementitious materials [24,25]. While H_2_S is the identified sulfur substrate in sewage environments, quantification of the concentrations of H^+^ and SO_4_^2−^ in direct contact with the cementitious materials is not possible.

The test from the University of Toulouse (the BAC test, for Biogenic Acid Concrete) presented in this study uses a soluble reduced sulfur source, tetrathionate (S_4_O_6_^2−^), to enable the quantification of the biological acid production in contact with the material, which is calculated from the sulfate production based on the assumption of a low proportion of sulfate trapped inside the cementitious matrix (controlled by sulfur mass balances in the leached solution) [26]. Tetrathionate is an intermediate sulfur species in the oxidation chain of sulfur into sulfate [17,26]. The sulfur source is dissolved in a nutritive solution and trickled onto the surface of the material in an optimized retention time of the feeding solution, creating a suitable environment for the production of sulfuric acid by the microorganisms in contact with the exposed surface and allowing the performances of the materials to be evaluated in only a 3-month period [4,6].

The validation of the representativeness of the test was demonstrated using two types of cementitious materials, blast furnace slag cement (BFSC) and calcium aluminate cement (CAC). The controlled environment led to the development of sulfur-oxidizing biofilm at the surface of the materials, which resulted in microstructural and chemical alteration of the cementitious materials [26]. The deterioration mechanisms and the performances of the two materials, as evaluated by the BAC test, were in accordance with the observations made in sewer networks exposed to H_2_S [12,17,26], and the impact of the material on the biofilm and the microbial activity was highlighted.

The BAC test established a simple and robust performance criterion (standardized leached calcium as a function of sulfate produced by the biofilm) which demonstrated its ability to distinguish and to highlight the differences in material performances regarding a biogenic acid attack in 3 months. The results obtained from five years of experiments using a wide range of materials were exploited to evaluate the repeatability and reproducibility of the test.

## 2. Materials and Methods 

### 2.1. Materials

#### 2.1.1. Cementitious Materials

The sewer environment is considered as an aggressive chemical acid environment (XA3) in the European Standard EN 206 + A1. Hence, different materials that are supposed to resist such environments were tested. The studied binders were ordinary Portland cement (OPC) (CEM I 52.5 N CE CP, LafargeHolcim, Le Havre, France) as the reference material, blast furnace slag cement (BFSC) (CEM III/B 32.5 N-LH/SR, Ciments Calcia, Rombas, France), composite cement (CEM V) (CEM V/A S-V 42.5 N PM-ES CP1, Ciments Calcia, Airvault, France), and calcium aluminate cement (CAC) (Secar 51, Imerys Aluminates, Fos-sur-Mer, France). The analyses of the chemical composition of the selected cements, presented in Table 1, were carried out by adapting the protocol from the European standard NF EN 196-2 [27]. The adapted protocol is detailed in Appendix A, and the detection limit, error, and the standard deviation of each compound are presented in Appendix B.

The reference material is a classic ordinary Portland cement which is made with 95% of clinker and 5% of gypsum. BFSC–corresponding to CEM III/B–is a slag-based binder composed of 71% slag, 29% Portland clinker and 6% calcium sulfate. CEM V is a composite cement made of Portland clinker mixed with slag and fly ash. Calcium aluminate cement is a special binder composed of alumina and lime as main oxides and silica, ferrite, and titanium oxide as secondary oxides.

Table 2 presents the mix design of the materials selected for the study. The mortar specimens were prepared in a standardized mortar mixer. The water to binder ratio was 0.4 for all specimens. However, the ratio of sand to binder was either 2.35 or 3.00 depending on the project requirements. The mixtures were cast in 4 cm × 4 cm × 16 cm molds and compacted using a jolting table.

Since the literature has reported different behaviors of materials against biogenic acid attack in sewer networks as a function of the nature of the aggregates [18,19,28,29,30,31,32,33,34], the purpose of using different types of aggregates (silica-based and calcium aluminate-based sand) in this paper was to demonstrate the ability of the test to highlight the influence of the aggregates on the performance of the material. The same binder (CAC or calcium aluminate cement) was used for CAC-S and CAC-A. On the one hand, the aggregates S are silica aggregates corresponding to the CEN standard sand according to the European standard NF EN 196-1 [35]. On the other hand, the aggregates A are synthetic aggregates with the same composition as CAC (Alag^TM^ aggregates).

After casting, while OPC, BFSC, and CEM V specimens were cured at 20 °C and 97% RH, CAC specimens underwent thermal curing. They were placed in a climatic chamber with a temperature gradient of 15 °C/h up to 70 °C, and then kept at 70 °C for 3 h. Twenty-four hours after casting, the specimens were demolded and kept in plastic bags at ambient temperature for 28 days. At the end of the curing period, the specimens were prepared following the procedure presented in Section 2.1.3 and were exposed to the BAC test for 13 weeks.

#### 2.1.2. Experimental Campaigns

Five different experimental campaigns were carried out in the frame of different research projects using the BAC test. Each campaign had specific purposes and objectives that led to the selection of specific materials, as reported in Table 3. Hence, the identifications (I), (II), (III), (IV), and (V), next to the name of the binder, correspond to the numbering of the different campaigns of exposure to the BAC test.

OPC mortar was systematically used in every campaign (except for campaign III, which was not taken into account for the PI calculations) as a reference material. The reference material was always prepared at LMDC using the same cement. However, the rest of the cementitious materials were prepared either by partners of the different projects or at LMDC.

#### 2.1.3. Preparation of Cementitious Specimens and Measurements before the Test

**Figure 1:**
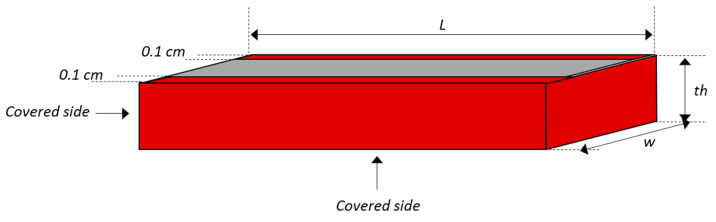
Scheme of the sawn mortar specimen with the side covered with an epoxy resin.

The recommended size of mortar specimens is 2 cm × 4 cm × 8 cm, typically obtained by sawing 4 cm × 4 cm × 16 cm mortar specimens into 4 equal parts. The dimensions (thickness (th), width (w), and length (L)) and the weight of the specimens were measured precisely to calculate the amount of calcium initially present in the specimen. An epoxy resin, EUROKOTE 48-20, was applied to the mortar specimens to cover 5 of the 6 surfaces, leaving only the top surface (corresponding to the surface from the core of the original 4 cm × 4 cm × 16 cm mortar specimen) exposed to the biodeterioration test as shown in Figure 1. Two thin lines (1 mm) of resin were also applied to the edges of the exposed surface in order to delimit the solution flow from upstream of the specimen to downstream and to preserve a reference of the initial thickness for further observations, by SEM for example (data not presented in this paper).

#### 2.1.4. Preparation of the Feeding Solution

The feeding solution contains a reduced sulfur source as well as the nutrients necessary for the development of microorganisms. Potassium tetrathionate (K_2_S_4_O_6_) (P2926, Sigma-Aldrich, Saint Louis, MO, USA) is the reduced sulfur source used in the BAC test. Table 4 and Table 5 present the composition of the mineral solution and the trace compound solution, respectively. The composition of the trace compound solution was not analyzed (nor measured) and was prepared manually by adding the different compounds to 1 L of deionized water. It was used as part of the feeding solution for microorganisms, and the trace compounds were not monitored over time. The trace compound solution was added as 5 mL for every 100 L of feeding solution.

Deionized water–or water of equivalent purity (pH 5–7.5) and conductivity < 0.1 mS.m^−1^–was used to fill a thermostatic tank with a minimum volume of 100 liters that contained a mixing system. The nutrients were dissolved salts prepared and stored in separate glass bottles at 4 °C to prevent any microbial growth. The nutrient salts and the reduced sulfur source were then added to the deionized water in the tank.

#### 2.1.5. Inoculum

Throughout the different testing campaigns, various activated sludges were used to inoculate the surface of the cementitious specimens. They were collected from different wastewater treatment plants at different times and were composed of diversified microbial populations. The surface of the specimens was inoculated only once, at the beginning of each experiment.

Activated sludges were collected from the sanitation system with solid particles in suspension in liquid solution. The centrifugation of 500 mL at 4000 rpm provided sufficient solid phase corresponding to the suspended organic matter of the activated sludge containing the microorganisms.

The repeatability of the results obtained with different sludges was validated in a previous study [17]. After the end of the testing period, samples of the biofilm were collected from the two CAC specimens that had been inoculated with two different activated sludges. The microbial populations in those biofilms were compared with another biofilm obtained from BFSC material inoculated with one of the activated sludges previously used for CAC as well as the initial consortium.

The analyses show that all initial inocula were composed mainly of heterotrophic (92%) and nitrifying (8%) bacteria, showing that sulfur-oxidizing bacteria were not initially detectable in the inocula. After the testing period, the biofilm analyses showed that sulfur-oxidizing bacteria were detected in the biofilm regardless of the material or activated sludge that was used. Heterotrophic bacteria were still identified in the biofilm at the end of the experiments but in a smaller quantity than in the initial consortium. This was in good agreement with the data reported for deteriorated concrete in sewer networks [36,37,38].

### 2.2. Experimental Setup and Testing Procedures

The biodeterioration test is suited to sewer environments where H_2_S is present and is applicable to any cementitious material (concrete for construction and/or mortars for rehabilitation of canalization systems), with or without admixtures (chemicals, biocide, etc.). Cementitious samples are first inoculated with a consortium (an activated sludge) and are then exposed to the trickling of a feeding solution containing a reduced sulfur source, tetrathionate (S_4_O_6_^2−^), and nutrients. In the presence of oxygen, sulfur-oxidizing bacteria in the consortium oxidize tetrathionate (S_4_O_6_^2−^) into sulfuric acid on the surface of the exposed cementitious samples.

The duration of exposure of the samples to the trickling solution is 3 months. The trickling solution is collected weekly downstream of each sample to analyze the concentration of calcium and sulfate ions and to measure the pH. In order to evaluate the resistance of mortars to biogenic sulfuric acid, the ratio of the cumulative leached calcium was standardized by the calcium initially present in the exposed materials per surface unit of exposed surface. The standardized leached calcium is presented as a function of the sulfate produced. The results presented in this paper come from 5 different campaigns which were involved in many national and European projects.

#### 2.2.1. Architecture of the Test

**Figure 2:**
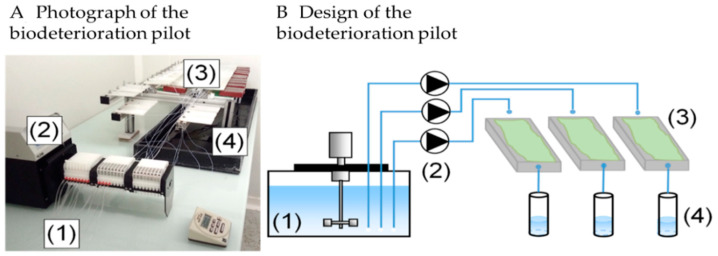
(**A**) Biogenic Acid Concrete (BAC) test pilot; (**B**) Schematic diagram of the BAC test [13].

The apparatus, presented in Figure 2, consisted of a feeding solution in a 100-liter thermostatic (4 °C) tank (1), connected to the specimens by plastic tubes (0.5–1.0 mm PTFE natural tubes) that transported the solution from the tank via flow pumps (regulated at 20 ± 5 mL/h) (2) and dropped it onto the top of the specimens (3). The specimens were placed on supports set at an angle of 5° to the horizontal in order to optimize the flow of the solution over the surface of the specimens. During the exposure period, samples of the leaching solutions were punctually collected downstream of the cementitious specimens and filtered immediately at 0.2 µm.

#### 2.2.2. Exposure Procedure

The first step of the exposure procedure was to inoculate the surface of the material with a microbial consortium collected from a wastewater treatment plant. This type of microbial consortium is known to be very diverse in terms of microbial population. Controlling the environment (feeding solution, exposed surface of the material, temperature and relative humidity) encouraged natural selection of the microbial activity and its adaptation to the surface of the material as in sewer environments [13,26].

The sludge was deposited delicately on the exposed surface of the cementitious materials using a brush and left to dry for 1 h so that it adhered well to the surface of the material.

**Figure 3:**
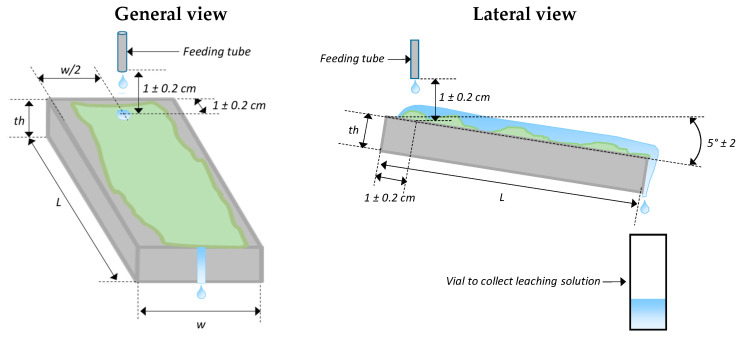
Scheme of one exposed cementitious specimen with the location of the feeding tubes and the collection tube for the leaching solution sample.

The second step consisted in placing the mortars on the supports while ensuring that the feeding solution droplets fell onto the exposed surface as described in Figure 3. The feeding tubes were placed 1 cm above the specimens to avoid contamination of the tubes by the biofilm. When the flow pumps were started, the first solution droplets were monitored until they fell off the material downstream. If it did not cover the entire surface of the material, the liquid film was gently spread over the surface with a brush (the biofilm was not yet developed at this stage, so there was no risk of distributing it). The pH of the solution was first measured for each specimen one hour after the beginning of the testing period.

#### 2.2.3. Collecting of the Leached Solution

Collecting the leached solution at the downstream end of the specimen over a given time duration permitted the solution flow over the specimens to be verified, the pH evolution in the trickling solution to be monitored during the exposure period, and the concentrations of leached cementitious calcium and leached sulfate (produced by microorganisms) to be quantified.

The flow pumps were then stopped and empty collection tubes (30 mL), previously weighed, were placed downstream of the specimens. The pumps were restarted for a sampling time of 60 ± 5 min. At the end of this time, the flow was cut off to take out the collection tubes before restarting it again. The exact leached solution flow over the exposed surface of the material at day “d_n_” (in mL/h) was obtained by the following equation:

Q_dn_ = (m_cn_ − m_c0_)/d_w_)/t_n_ (1)

with Q_dn_: flow of the leached solution at day “d_n_”, expressed in mL/h; m_cn_: weight of the collection tubes filled with the leached solution, expressed in g; m_c0_: weight of the empty collection tubes, expressed in g; d_w_: water density, expressed in g/L; t_n_: collection time, expressed in hours.

#### 2.2.4. Analyses of the Leached Solution

The pH was measured with a pH meter directly after collection of the leached solutions in the tubes with a precision of 0.01 pH unit for each sample. The solutions were then filtered to 0.2 µm to eliminate all parts of biofilm, microorganisms, and particles that could have fallen into the tubes during the collection period. The liquid samples were stored at 4 °C to avoid proliferation.

The concentrations of calcium in the leached solutions were determined using inductively coupled plasma with optical emission spectrometry (ICP–OES) [39,40,41]. The used ICP–OES equipment was an Optima 7000 DV with a Meinhart nebulizer. The generator power was 1450 W. The flows of argon and air (auxiliary gas) were 15 and 0.2 L/min, respectively. The flow at the entry of the nebulizer was 0.6 L/min.

Firstly, standards of calcium were prepared at 0.5, 1.0, 5.0, 10.0, 20.0, 50.0, and 100.0 mg/L. Secondly, the solution samples were diluted with a factor of 10 to reduce the calcium concentrations in the leached solutions into the standards concentration range. ICP–OES is more stable when analyzed liquid matrices are acid; hence, the standards and the diluted leached solutions were prepared using ultrapure water with 2 vol.% of nitric acid.

The concentrations of sulfate in the leached solutions were measured using high-performance ion chromatography (HPIC) (Dionex ICS-3000). In our conditions, because of the microbial sulfur-oxidizing activity in the biofilm on the surface of the materials, the leached solutions contained different types of polythionates, which disturbed the identification and quantification of the amount of produced sulfate using usual techniques. A dedicated method was thus developed to measure the sulfate concentrations after separating the sulfate from the other polythionates. In particular, the pre-column and column were a Dionex AG11 and an IonPac AS11-HC, respectively. The column flow was of 1.5 mL/min. The gradient of eluent (KOH) was between 1 and 60 mM and the column temperature was 30 °C. A suppression system (AERS 500 4 mm) was used to increase the sensitiveness and to reduce the background conductivity of the eluent.

Sulfate standards were prepared at 0.1, 0.5, 1.0, 5.0, 10.0, 20.0, and 50.0 mg/L. The leached solution samples were diluted with different factors during the testing period. In the early stages of the test, the concentration of sulfate was known to be very low; therefore, the dilution factor was 5. However, as the intensification of the attack was observed via the pH evolution of the leached solution, the dilution factor increased up to 20.

More information about the methods, equipment, and standard solution preparation used for the chemical analyses are presented in Appendix C.

#### 2.2.5. Presentation of the Leaching Results of the BAC Test

The concentration of sulfate in the leached solution is an indicator of the biogenic production of sulfuric acid over the exposed surface of the cementitious materials. Therefore, the objective here was to express the cumulative leached cementitious ion per the initial content of the cementitious ion in the exposed material per square meter of exposed surface as function of the cumulative amount of sulfate produced. In what follows, leaching of calcium will be used as an example.

The analyses of the leached solutions provided the calcium concentration during the period of exposure to the BAC test of the different materials. Thus, the flux of leached calcium for each specimen could be calculated following Equation (2):

F(Ca^2+^)_dn_ = Q_dn_ × [Ca^2+^]_dn_ × 10 ^−3^ (2)

with F(Ca^2+^)_dn_: flux of leached calcium ions at day d_n_ (in mol/h); Q_dn_: flow of leached solution at day d_n_ (in mL/h); [Ca^2+^]_dn_: concentration of leached calcium measured in the leached solution at day d_n_ (in mol/L).

The same calculation was carried out at day d_n+1_ to obtain F(Ca^2+^)_dn+1_ using the amount of calcium leached between day d_n_ and day d_n+1_, calculated following Equation (3):

Tot(Ca^2+^)_dn+1−dn_ = ((F(Ca^2+^)_dn+1_ + F(Ca^2+^)_dn_)/2) × (d_n+1_−d_n_) (3)

with Tot(Ca^2+^)_dn+1–dn_: cumulative leached calcium between day d_n+1_ and day d_n_, expressed in mole; (d_n+1_–d_n_): the time between day d_n+1_ and day d_n_, expressed in days.

Finally, the results of the cumulative cementitious calcium were standardized by the initial content of calcium in the exposed material and the exposed surface. Thus, the exposed surface was evaluated based on the dimensions (Section 2.1.3) measured prior to the exposure of the specimens to the BAC test. The exposed area was calculated using Equation (4):

S = L × w(4)

with L: the length of the exposed specimen, expressed in m; w: the width of the exposed specimen, expressed in m.

STot(Ca^2+^) = Tot(Ca^2+^)_dn+1−dn_/Tot^init^(Ca^2+^)/S, expressed in molCa/molCa/m^2^ (5)

Finally, the performance indicator (PI) translates the resistance of material “X” to biogenic acid attack in sewer conditions compared to the resistance of ordinary Portland cement mortar (OPC). However, in order to compare the resistance of a material to that of the reference material, the ratio should be calculated for the same amount of measured sulfate. Thus, a linear model was fitted to the experimental results, with the equation:

STot_x_ = A × [SO_4_^2−^] + B (6)

where A and B are constants calculated by the least squares method. Then, the model was applied to a wide range of sulfate concentration for the different materials. Consequently, the PI is calculated as follows:

PI_x_ = 100 × (STot_x_/STot_OPC_) expressed in % (7)

In order to present the results of the leached cementitious calcium as a function of the cumulative sulfate, the total leached sulfate was evaluated using the same calculation as for the cementitious cations.

#### 2.2.6. Maintenance of the BAC Test Pilot during the Exposure Period

The feeding solution passes through the connecting plastic tubes before falling off on top of the material. Such tubes are not sterile; hence, the risk of developing a biofilm inside the plastic tubes–which could go up to the feeding solution tank–is not null. The colonization of the tubes could result in the production of acid not in contact with the material, and, thus, bias the results of the test. In order to prevent microorganisms flourishing in the tubes, the feeding solution was renewed and the tank containing it was cleaned using bleach every 2 weeks. Before the tank was cleaned, 5 liters of the feeding solution was collected in an adequate clean container, and the tubes were put into the newly filled container to ensure continuous supply of the solution to the exposed materials during the cleaning of the feeding tank. The plastic tubes were renewed regularly, typically every 2 weeks, to stop any microbial growth outside the exposed surface of the material.

## 3. Results

### 3.1. pH Evolution and Acid Production

**Figure 4:**
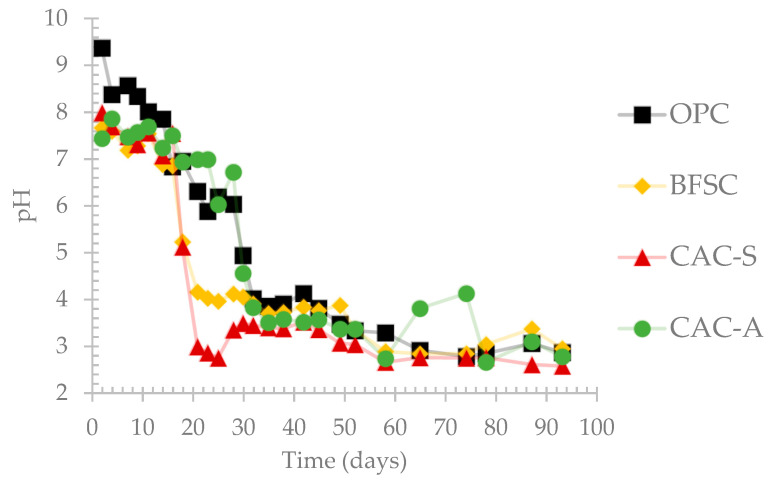
Evolution of the pH of the leaching solution during the exposure period.

The evolution of pH of the leached solutions is shown in Figure 4. The progressive development of the biofilm on the surface of the materials led to the production of sulfuric acid, which induced a decrease in the pH of the leaching solution. This decrease occurred in four phases: (i) one day after the start of the exposure period, when the pH was relatively high for all materials, around 8 for non-Portland cement-based materials and 10 for Portland cement-based materials. The first phase lasted for 15 days for all materials. (ii) From 15 days and depending on the nature of the material, a strong pH decrease to acidic values (<4) was observed, caused by production of the acid by SOB. (iii) From 30 days and up to 50 days, the pH values of the leaching solutions were between 3 and 4. (iv) The fourth and last phase was characterized by a relatively constant pH, around 2.5, from day 50 until the end of the test.

The pH evolution highlighted the differences in the time before acidification between OPC, BFSC, and CAC. OPC had the highest buffer effect, followed by BFSC and CAC. However, the use of alumina-based sand instead of silica-based sand provided the material with a greater neutralizing capacity [13]. CAC-A showed almost the same decrease of pH of the leaching solution as OPC did. The pH of OPC and CAC-A reached acidic values after 30 days, compared to 20 and 25 days for CAC-S and BFSC, respectively. However, for phases 3 and 4, all materials remained at the same range of pH and the difference between the materials was negligible.

**Figure 5:**
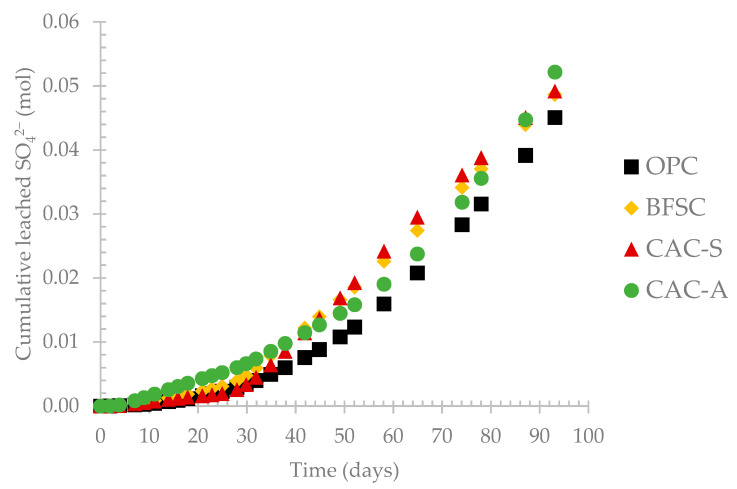
Evolution of the sulfate concentration in the leaching solution during the exposure period.

Figure 5 presents the evolution of sulfate production measured in the leached solution. For the early stages of exposure to the BAC test, a small amount of sulfate was quantified in the leached solution, corresponding the first phase (i) of the pH evolution. Phase (ii), corresponding to the decrease of pH from 7 to 4 due to the activity of NSOB as reported in the literature, showed a slight increase of sulfate concentration in the leaching solutions [6]. The progressive growth of acidophilic bacteria started around pH 4, where the production of sulfuric acid increased significantly [6,7,36,42]. Between 30 and 50 days (phase (iii)), the pH condition limited the activity of the neutrophilic bacteria and led to a transition period where NSOB and ASOB could cohabit [6,7,43]. This period showed a progressive increase in acid production, corresponding to the increase in leached sulfate in the same period. From 50 days, the amount of sulfate increased rapidly in the leached solution–corresponding to the fourth phase of the pH evolution–due to the acidification of the medium, leading to the development of more acidophilic SOB and their high activity rate at low pH (2.5–3) [3,6,7,24,36,43,44].

### 3.2. Standardized Calcium Leaching

**Figure 6:**
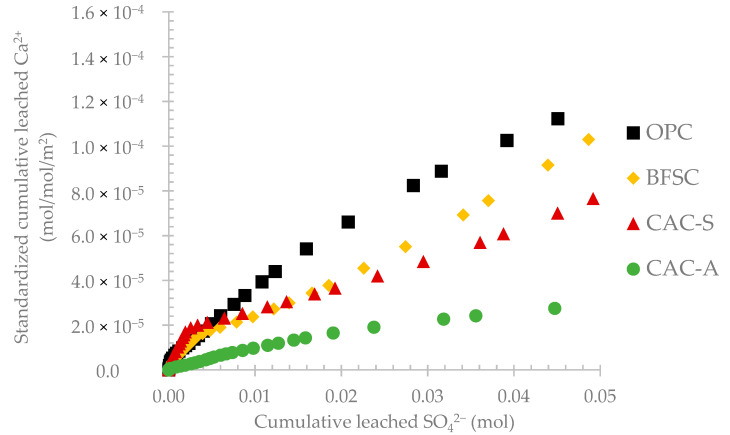
Evolution of the standardized leached calcium as a function of the leached sulfate during the exposure period.

Figure 6 presents the evolution of standardized leached calcium as a function of produced sulfate. Except for very low amounts of sulfate (<0.01 mol), the plots are substantially linear as a function of produced sulfate. Relatively to their low initial amount of calcium, CAC materials released less Ca^2+^ compared to OPC and BFSC. CAC-A material showed the lowest relative Ca leaching followed by CAC-S, BFSC, and then OPC.

The evaluation of standardized calcium leaching highlighted the performance of each material respectively to the amount of sulfate produced by the bacteria. Although the sulfate amount was relatively similar for all the materials, the resistance of each material was different. Moreover, the pH of the leaching solution showed very similar evolution for OPC and CAC-A, but the leaching of calcium was completely different.

The results obtained from the BAC test are in accordance with the data in the literature reporting the exposure of cementitious materials in real sewers. Reducing the amount of OPC in a binder by substituting with supplementary cementitious materials (such as slag) could improve the resistance of the material to biogenic acid attack [13]. Moreover, CAC-based materials showed better resistance to biogenic acid attack in sewer conditions than OPC and BFSC [12,13,17,19,28,45,46,47]. The difference in the relative amount of leached calcium between calcium aluminate-based and Portland-based cementitious materials increased significantly from 0.02 mol of leached sulfate, i.e., day 50 (pH drop to between 2.5 and 3) of exposure to the BAC test. In addition, replacing inert aggregates (silica-based) with reactive aggregates (calcium aluminate-based) resulted in better performance of the CAC material [19,30,47].

### 3.3. Calcium Leaching from the Same Campaign

**Figure 7:**
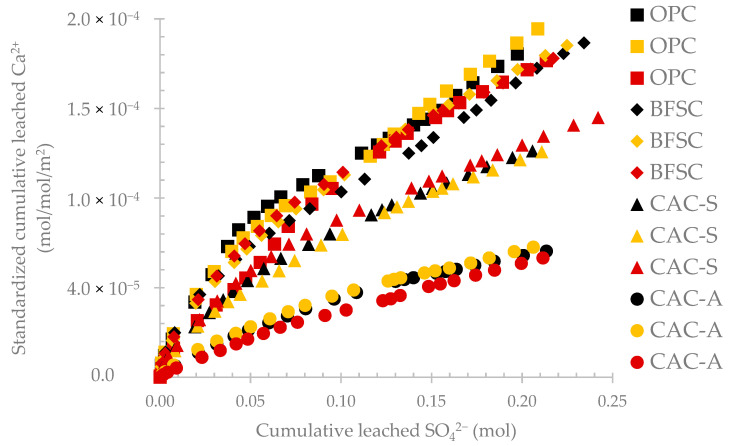
Evolution of standardized leached calcium per square meter as a function of the cumulative leached sulfate for the same testing campaign on four different materials

Figure 7 presents the standardized leached calcium per square meter of the exposed area of materials as a function of the cumulative leached sulfate in the same testing campaign on four different materials: OPC, BFSC, CAC-S (silica-based sand) and CAC-A (alumina-based sand). During the active deterioration phase (iv), the 3 specimens of each material showed very similar behavior in terms of calcium leaching. The variation in the standardized cumulative calcium for each material was assessed and the error margins are reported in Table 6. The maximum error margin represents the highest positive difference between the 3 specimens and the average of these specimens while the minimum error margin represents the lowest negative difference between the 3 specimens and the average of these specimens. The variation was less than 13% for all materials, which still indicated a good repeatability of the results obtained from the BAC test in the same campaign on 3 specimens of the same material.

### 3.4. Calcium Leaching from Different Campaigns

**Figure 8:**
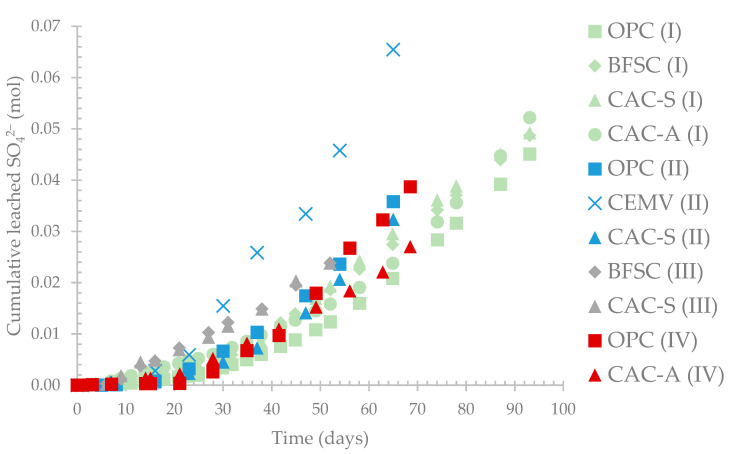
Cumulative leached sulfate from different testing campaign using the BAC test.

Figure 8 presents the cumulative leached sulfate from different materials and from 4 different testing campaigns (noted I, II, III, and IV) using the BAC test. The different colors distinguish the different campaigns, and the symbols correspond to the different types of materials. Regarding campaigns I (green) and III (grey), the amounts of cumulative leached sulfate were similar for the 4 different materials, while for campaign II (blue), the measured sulfate for CEM V was different from that of the other materials. Campaign IV showed some differences in the sulfate starting from 50 days, which corresponds roughly to phase (iv) of the pH evolution. The production of sulfuric acid on a material’s surface is dependent on the activated sludge (inoculum)–which was not the same for all the campaigns–and on the physicochemical properties of the cementitious material which could accelerate or delay the development of the biofilm.

**Figure 9:**
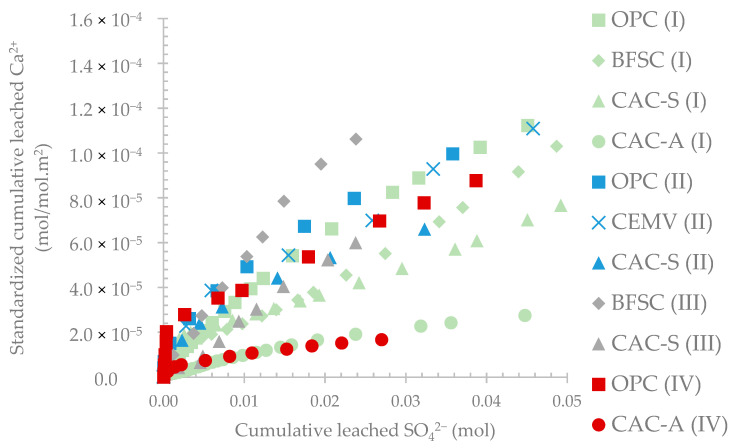
Evolution of the standardized cumulative leached calcium per square meter in function of the cumulative leached sulfate in the leached solution.

Figure 9 presents the standardized leached calcium per square meter of exposed area as a function of the cumulative sulfate measured in the leached solution for each material. When certain results obtained in one campaign are compared to some obtained in other campaigns, some uncertainty can be observed. For instance, CAC-S from campaign II (blue) and BFSC from campaign I (green) released similar relative Ca^2+^ amounts. Moreover, BFSC from campaign III (grey) released significantly more relative Ca^2+^ than BFSC from other campaigns. Furthermore, CAC-S curves overlap the zone where Portland-based materials are located. Therefore, the durability indicator should integrate the reference material tested in each campaign, the nature of which is always the same: a CEM I mortar (see Section 2.1.2).

The purpose of using the reference material was to classify the performance of the tested materials independently of the considered campaign. For a given campaign, the PI (performance indicator) of each material was calculated as the standardized cumulative leached calcium values divided by the standardized cumulative leached calcium of the reference material (OPC).

**Figure 10:**
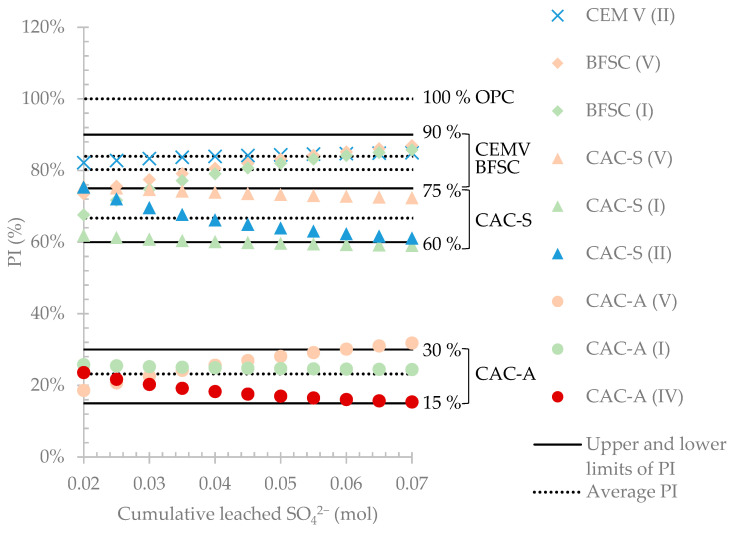
Evolution of the standardized cumulative leached calcium per square meter relative to the ordinary Portland material in the same campaign as function of the cumulative leached sulfate in the leached solution.

When the amount of sulfate was very low, the biofilm was not yet developed; hence, the PI of each material was determined for leached calcium concentrations between 0.02 and 0.07 mol of cumulative leached sulfate. Figure 10 presents the relative standardized leached calcium per square meter as a function of cumulative sulfate concentration measured in the leached solution together with the calculated PI intervals for all materials.

The reference materials (OPC) of each campaign had a PI of 100%, which means that the materials found to have PIs above 100% did not perform as well as the reference material and materials with PIs lower than 100% performed better than the reference material. The PIs of CEM V and BFSC were 84% and 80%, respectively. These materials could be considered to have relatively similar performances, with a slight increase in resistance (depending on the nature of the materials) compared to Portland cement. CAC materials–with silica- and calcium aluminate-based sands–performed better than Portland materials against the biogenic attack. The PIs of CAC-S and CAC-A were 67% and 23%, respectively, showing the strong influence of the reactive aggregate on the performance of the mortar.

The reproducibility of the results was assessed using the results for the same material from different campaigns. Although it was highly unlikely that exactly the same material would have the same PI in different campaigns due to the experimental variations, a confidence interval (CI) was calculated based on the variation observed from the experimental data and reported in Table 7. The CI gives a zone in which the materials can be identified according to their performance.

## 4. Discussion

### 4.1. Bacterial Settlement and Microbial Activity

The development of the biofilm on the surface of the cementitious materials tested in the BAC test occurs due to a natural selection of sulfur-oxidizing bacteria from the activated sludge. Such selection was ensured by the use of tetrathionate as the reduced sulfur source and confirmed by the evolution of the pH of the surface of the materials.

The four different stages of the pH evolution were consistent with the observations found on cementitious materials exposed in situ [12,32,48]. In the early stages of the biogenic attack, the production of sulfuric acid was low due to the low activity of sulfur-oxidizing bacteria in the inoculum [9,13,26]. However, the sulfate concentration in the leaching solutions increased after 10 days of exposure to the BAC test, which corresponded to the development of neutrophilic SOB. During this stage, the production of acid on the surface of the materials resulted in the decalcification of calcic hydrates–such as CH and C-(A)-S-H for Portland-based materials, and C_3_AH_6_ and CAH_10_ for CAC-based materials–and the release of OH^−^ [12,13,33,48].

Although no major disparity was found in the amount of sulfate produced on the surface of the different materials in the same campaign, the reactivity of the cementitious phases differed depending on the nature of the materials. The availability of calcic phases, which are the first to react with the acid, varies from one material to another. For example, CH and C-S-H constitute roughly 50–60 wt.% of the hydrated matrix of an OPC material, while C_3_AH_6_ forms approximately 40 wt.% of that of CAC material [13,17,32,49]. In addition, calcic phases in Portland cement appears to be more reactive than calcic phases found in CAC cement [30,33]. Therefore, the amount and reactivity of the calcic phases will influence the surface pH and, hence, influence the development of acidophilic bacteria.

Since released hydroxide ions could be considered as the main factor responsible for the neutralization capacity of the material, the higher amount of OH^−^ released from calcic hydrates for Portland cement–compared to calcium aluminate cement–could explain the delay in the acidification for the PC binder [12,13,33]. Blending OPC with slag (BFSC material) reduced mainly the amount of Portlandite, thus inducing a decrease in the buffer capacity of the material. Moreover, the influence of the nature of the aggregates on the buffering capacity of the material was also observed on specimens exposed to the BAC test [19,29]. Replacing the non-reactive silica-based aggregates (CAC-S) by reactive calcium aluminate-based aggregates (CAC-A) increased the amount of OH^−^ ions in the binder. Thus, the buffer effect of the material was extended in time and led to higher neutralization capacity.

A previous study with the BAC test showed that the calcium leaching from the materials was linear as function of time during the whole testing period (except for very low amounts of cumulative sulfate <0.01 mole) [13]. Thus, the deterioration process was mainly controlled by a surface reaction leading to a dissolution mechanism on the surface of the specimens with a small contribution of diffusion phenomena.

The acidification of the surface of the CAC-S and BFSC occurred after 20 days of exposure to biogenic acid attack, while for OPC and CAC-A, it took 30 days for the pH to reach acidic values. Such acidification resulted in the growth of ASOB accompanied by the production of a significant amount of acid. While the development of ASOB in sewer networks takes from several months to several years, as reported in the literature, the BAC test was able to reach the active deterioration phase of the materials in sewer conditions after only 1 month [32,48,50].

### 4.2. Performance Indicator (PI)

In the literature, the durability of cementitious materials exposed to sewer conditions has mainly been assessed by measuring the mass loss. However, mass loss can misrepresent the effective durability because it is not unknown for pieces to come loose during the preparation of the material and the precipitation of secondary phases could disturb this measurement; the literature has not reached a consensus on the protocol for measuring this mass loss (brushing, ultrasound waves, immersion in solution, etc.).

Since the dissolution and the precipitation of the cementitious phases were bounded by thermodynamic equilibrium and chemical conditions (concentration in the interstitial solution and pH of the pore solution), monitoring of the pH, sulfate production by the microorganisms, and cementitious cations released in the leached solution is essential to evaluate the durability of the materials. Such quantification allows the process of material deterioration to be monitored as function of pH and the amount of acid that was in direct contact with the material during the period of the experiment to be quantified. Moreover, the standardization of the leached calcium as a function of cumulative produced sulfate allows an association to be found between the quantity and the kinetics of the calcium leached by the acid attack as well as permitting a comparison of the results from different campaigns.

The materials tested using the BAC test showed different performances according to their chemical/mineralogical nature. PC and BFSC matrices are composed of hydrates mainly in the calcium silicate system, while CAC is composed of hydrates from the calcium aluminate system [51]. The main parameter influencing the resistance of the materials, as suggested in several studies, is the mineralogical composition [13,30,32].

When the cementitious hydrates are attacked by acid, decalcification and dissolution of these phases take place, leading to the release of calcium into the leached solutions. The classification of the materials according to their resistance to biogenic attack in sewer conditions was assessed by the parameter of calcium released relative to the amount of initial calcium in the material. However, in order to determine an absolute performance of these materials independently of the campaign, an indicator was developed–named the performance indicator (PI)–to compare the resistance of the material under test to that of the reference material (OPC) used in any campaign. Using the PI, the classification of the materials was (from the lowest to the highest resistance): CEM V (84%) < BFSC (80%) < CAC-S (67%) < CAC-A (23%).

Data from the literature confirm the results obtained from the BAC test in terms of classification of materials. When Portland cement is attacked by acid, the main phases (CH and CSH) of this binder decalcify, leaving a porous silica gel that could lead to penetration of the acid to deeper zones [13,15,25,29,32]. Since the biogenic acid attack in sewers is a combined attack of acid and sulfate, the penetration of sulfate triggers local precipitation of secondary expansive phases, mainly identified as gypsum and ettringite [13,17,32,48,52]. The swelling caused by the precipitation of such phases results in internal stresses and cracking of the hardened cementitious material, leading to more penetration of the acid [17,20,53]. Blending slag with PC (BFSC) lowers the total content of calcic hydrates likely to react with the acid, thus improving the resistance of the binder [13,32]. However, the resistance of such binders depends strongly on the amount of slag in the mixture. In addition, CAC-based materials performed better than PC materials in both laboratory and in situ conditions [12,13,17,19,30,45,48,54]. The better resistance of CAC was associated with the lower reactivity of its initial and decalcified phases [13,33]. The literature has reported that decalcification of C_3_AH_6_ leads to a precipitation of AH_3_ gel. AH_3_ phases are known to be chemically stable over a wide range of pH (from ~10 to ~4 depending on the Al concentration) which improves the resistance to the acid even in acidic environments [12,33,54]. Moreover, in a highly acidic medium, the alumina hydrate dissolves and neutralizes more acid at later stages of the biogenic attack [33]. CEM V was found to perform slightly better than PC. Such performance could be attributed to the low initial Portland cement content and the incorporation of different supplementary cementitious materials. C-(A)-S-H are likely to form in such binders which could have a slightly better resistance than C-S-H phases traditionally found in hydrated Portland cement. While the literature shows that CEM V can have a resistance similar to that of PC when exposed to biogenic acid attack in sewer networks, there is currently no clear explanation or hypothesis on the reasons for its performance [45,55].

Finally, the PI has demonstrated its capacity to discriminate between the performances of aggregates of different natures. The PI showed that calcium aluminate-reactive aggregates performed around 3 times better than inert silica aggregates. Literature data show that using calcium aluminate-based aggregates with CAC binder significantly improves the durability of the designed material [18,19,29,30,31,32,33,34]. Nevertheless, it should be noted that calcium aluminate aggregates are synthetic aggregates composed of the same mineralogical anhydrous phases as the CAC. Hence, compared to standardized silica-based aggregates, calcium aluminate aggregates could have better adhesion and cohesion with CAC cementitious matrix which could contribute to their better performance.

### 4.3. Repeatability and Reproducibility Assessment

The results presented in this paper were acquired over 5 years. Five different testing campaigns using the BAC test were carried out in different research projects. Ordinary Portland cement was used systematically as a reference material for all campaigns.

The development of biofilm on the cementitious materials occurred without any outside intervention. The bacteria–present in the activated sludge–were selected naturally based on the available substrate, tetrathionate (S_4_O_6_^2−^) in this case. Hence, the evolution of the microbial activity can differ slightly from one specimen to another in the early stages of the test. Nevertheless, as seen from the pH evolution and the production of sulfate, this phase was characterized by low production of acid and low activity of sulfur-oxidizing bacteria. The active deterioration phase came later when the biofilm developed on the surface of the specimens.

The repeatability of the results was determined by the same operator repeating the same measurement (Ca^2+^ concentration in the leached solution) at the same time for 3 different specimens of the same material (same formulation, same casting parameters and same inoculation). The results demonstrated the ability of the test to give very similar results with relatively small error margins. It should be noted that the values of the relative standardized calcium were very small (1.0 × 10^−4^ magnitude), so any slight variation could lead to a relatively high error margin. Nevertheless, having error margins of less than 13% reflects very good repeatability in terms of material performances. Moreover, the kinetics of calcium leaching from the cementitious matrix were very similar.

The reproducibility of the results was determined by the variance between the results obtained by different operators conducting the same test at different times using the same equipment in the same laboratory. The variability of the results from one campaign to another was mainly linked to the development of the biofilm. The decision to use different activated sludges during the different campaigns was made in order to demonstrate how representative the test was of the actual deterioration phenomena in sewer networks. Although the nature of the sludge could differ depending on when and where it was collected (climate, industrial or residential area, etc.), the behavior of the material toward the acid attack did not vary. In order to assess the behavior of the materials toward the different bacterial consortia, a confidence interval was calculated to determine different zones in which the results obtained experimentally should be found according to each material. Small variabilities were observed in the results from the different campaigns; however, these variabilities remained in the areas identified for each material (reported in Table 7).

## 5. Conclusions

The paper has presented the full description of the Biogenic Acid Concrete (BAC) test which was designed to evaluate the performance of cementitious materials to biogenic sulfuric acid attack in sewer environments. The representativeness of the BAC test with respect to the in situ phenomena was validated in previous studies [17,26,56]. The results presented here were collected over five years of experimental campaigns, carried out in different projects with different materials.

The BAC test used a source of soluble reduced sulfur (tetrathionate) to quantify the amount of acid produced by bacteria in contact with the material’s surface. It is safer to conduct experiments using tetrathionate instead of hydrogen sulfide because of the toxicity of H_2_S at high concentrations. In addition, a precise quantification of the reactivity of the materials was obtained by monitoring the amount of calcium leached during the testing period. Finally, the test was able to discriminate the performances of different materials over a period of only 3 months.

The biodeterioration results found with the BAC test were globally in accordance with in situ and laboratory data from the literature. The selection of sulfur-oxidizing bacteria–neutrophilic and then acidophilic–was successively achieved by using tetrathionate as the reduced sulfur substrate in the feeding solution. The microbial activity induced the production of sulfuric acid, which decreased the surface pH to about 2.5. The biogenic acid attack led to the decalcification and dissolution of the main cementitious phases, which resulted in the release of calcium ions in the leaching solutions.

The reactivity of the phases probably controlled the dissolution of the cement matrix and, thus, the resistance of the materials submitted to the BAC test in aggressive conditions. The BAC test was able to represent the significant influence of the buffer capacity of the materials on microbial growth. The evolution of the pH differed according to the chemical and/or mineralogical nature of the materials.

The capacity of the BAC test to differentiate between the performances of cementitious materials in a relatively short time (3 months) was emphasized by selecting a wide range of materials with different chemical and mineralogical compositions. Simply by comparing the leaching kinetics and the total amount of calcium leached relative to the calcium initially present in the cementitious material, the contrast in the resistance of the materials, with respect to their nature, was highlighted.

The performance indicator (PI) was developed in order to classify the cementitious materials according to their resistance to biogenic sulfuric acid attack in comparison with the resistance of Portland cement. This classification was in accordance with the observations and results obtained in various studies carried out in situ in several parts of the world and reported in the literature.

The repeatability of the results was demonstrated by monitoring the leaching kinetics and the total amount of calcium ions leached within the same campaign on several specimens of the same material. Likewise, the reproducibility of the results was assessed over various testing campaigns. This led to the development of a confidence interval around the PI values, where all materials of the same nature were located inside the interval. The comparison between different experimental campaigns was made possible by the standardization achieved by comparing the results obtained to the performance of OPC material (reference material).

## Figures and Tables

**Table 1:** Chemical composition (in wt.%) of the different cements used in this study.

Reference	CaO	SiO_2_	Al_2_O_3_	Fe_2_O_3_	SO_3_	MgO	TiO_2_	K_2_O	Na_2_O	P_2_O_5_	Mn_2_O_3_	Ignition Loss
OPC	65.57	19.97	5.51	2.77	3.97	1.16	0.25	0.25	0.12	0.38	0.14	-
BFSC	49.13	29.60	9.41	1.21	3.22	4.69	0.52	0.59	0.24	0.06	0.27	1.02
CEM V	46.36	30.03	11.15	3.59	2.80	2.75	0.58	1.16	0.22	0.61	0.12	2.05
CAC	37.93	5.08	52.13	1.63	0.04	0.31	2.06	0.36	0.08	0.11	0.01	-

**Table 2:** Description of mortar compositions.

Reference	Cement	Standardized Designation	Aggregates	W/B
OPC	Portland cement	CEM I	Standardized silica sand	0.40
BFSC	Portland cement	CEM III		0.40
CEM V	Portland cement	CEM V		0.40
CAC-S	Calcium aluminate cement	-		0.40
CAC-A		-	Calcium aluminate-based sand	0.40

**Table 3:** The experimental campaigns and the cementitious materials tested in each campaign.

Exp. Campaigns	I	II	III	IV	V
Tested cementitious materials	OPC	OPC	-	OPC	OPC
	BFSC	BFSC	BFSC	-	BFSC
	-	CEM V	-	-	-
	CAC-S	CAC-S	CAC-S	-	CAC-S
	CAC-A	-	-	CAC-A	CAC-A

**Table 4:** Composition of the feeding solution.

Compound	Concentration	Unit	Salt
S_4_O_6_^2−^	158.86	mgS-S_4_O_6_^2−^/L	K_2_S_4_O_6_
N-NH4^+^	3.93	mgN-NH_4_^+^/L	NH_4_Cl
P	1.71	mgP/L	(Na(PO_3_))_3_
Mg^2+^	0.18	mgMg^2+^/L	MgCl_2_, 6H_2_O
Mn^2+^	0.03	mgMn^2+^/L	MnCl_2_
Fe^3+^	0.02	mgFe^3+^/L	FeCl_3_, 6H_2_O

**Table 5:** Composition of the trace compound solution.

Compound	Concentration (g/L)
H_3_BO_3_	0.30
CoCl_2_, 6H_2_O	0.20
ZnSO_4_, 7H_2_O	0.10
Na_2_MoO_4_, 2H_2_O	0.03
CuSO_4_, 5H_2_O	0.01
NiCl_2_, 6H_2_O	0.02

**Table 6:** Error margins for materials tested in the same campaign.

Property	Materials	Error Margins (%)	
		Maximum	Minimum
Standardized cumulative leached Ca	OPC	+10.96	−8.19
	BFSC	+2.34	−2.34
	CAC-S	+5.70	−11.14
	CAC-A	+12.25	−8.19

**Table 7:** Confidence interval for tested materials from four different campaigns.

Property	Materials	PI (%)	Confidence Interval (%)	
			Positive	Negative
Standardized cumulative leached Ca	OPC	100	-	-
	CEM V	84	-	-
	BFSC	80	84	74
	CAC-S	67	74	60
	CAC-A	23	28	18

## Data Availability

Data is contained within the article or Appendix.

## Appendix A

An adapted protocol from the European standard NF EN 196-2 was used for chemical analyses of cements [27]. The analyses had 3 components: the first component concerned the total element content (Si, Al, Ca, Mg, Mn, Fe, Na, K, Ti, P, Cr, Sr, etc.) which was assessed via the fused cast-bead method followed by an acid attack and an analysis by wet chemistry. The concentration of elements was further quantified by ICP–OES. The anhydrous cement was slightly crushed to <80 µm, and 0.2 g of the powder was put into a vitrified graphite crucible. A 1.2 g sample of lithium borates (25% metaborate/75% tetraborate) was added to the cement powder and mixed a homogenous mix. The graphite crucible was inserted into a graphite sheath with a cover and was put into a muffle furnace at 1100 °C for 30 min. The graphite crucible was taken out of the furnace and was put aside for 5 min to cool down before inserting a magnetic bar into the crucible. Consequently, the set (crucible + magnetic bar) was put into a solution made of 100 mL of ultrapure water + 5 mL of nitric acid (HNO_3_ plasma pure). Continuous stirring using the magnetic bar was carried out until the total dissolution of the beads. Once the beads were completely dissolved, the liquid solutions were filtered into a vial using quantitative adequate filters, and the volume of solution was adjusted to 250 mL with ultrapure water. The second component concerned the quantification of oxyanions fluoride, sulfate, nitrate, and phosphate in the cementitious matrix, which were quantified via high-performance ion chromatography (HPIC) in a solution prepared via acid attack of the cement powder sample, since the analysis of the solution issued from the fused cast-bead attack was too concentrated in lithium. The cement was slightly crushed to <80 µm and 1 g of the powder was put into a hydrochloric acid solution (95 mL of ultrapure water + 5 mL of HCl plasma pure). The mix was boiled for 2 min with continuous stirring using a glass spatula. The liquid mix was filtered into a vial using quantitative adequate filters, and the volume of solution was adjusted to 250 mL with ultrapure water. The third component concerns the ignition loss, which was carried out following the European Standard NF EN 196-2 (ranking index: P 15-471-2).

Moreover, a reference cement provided by the Bureau of Analysed Samples LTD (BCS-CRM No.353), the composition of which is certified (British chemical standard certified reference materials), was used systematically to calibrate the analyses. The chemical composition of this reference cement was obtained from 9 independent analysts [57].

## Appendix B

Table A1 presents the elemental chemical composition of the cements used in this study. However, the chemistry of cements is a chemistry of oxides where the composition of cements is expressed in oxides of the elements (Ca, Si, Al, Fe, Mg, Mn, Ti, K, Na etc.). The oxide composition results from calculations from the elemental composition by a molar mass ratio of the corresponding oxide (CaO, SiO_2_, Al_2_O_3_, Fe_2_O_3_, MgO, Mn_2_O_3_, TiO_2_, K_2_O, Na_2_O, etc.). The complementary to 100 wt.% comprises the content of oxygen and the loss on ignition (provided Table 1).

**Table A1:** Elementary chemical composition (in wt.%) of the different cements used in this study.

wt.%	Ca	Si	Al	Fe	SO_4_	Mg	Ti	K	Na	P	Mn
OPC	46.87	9.34	2.91	1.94	4.76	0.70	0.15	0.21	0.09	0.17	0.10
BFSC	35.12	13.84	4.96	0.85	3.86	2.83	0.31	0.49	0.18	0.03	0.19
CEM V	33.14	14.04	5.88	2.51	3.36	1.66	0.35	0.96	0.16	0.27	0.08
CAC	27.11	2.38	27.50	1.14	0.05	0.19	1.23	0.30	0.06	0.05	0.01

Table A2 presents the detection limit of the used machines (ICP–OES and HPIC), the error for each compound as well as the standard deviation of the results. The detection limit for each compound was obtained from the characteristics of the machine (ICP–OES and HPIC). A reference cement was used to determine the error for each compound between the analyzed cement and the theoretical values which were obtained from the certificate of analysis of the reference cement. Finally, the reference cement was analyzed five times to determine the mean and the standard deviation of the results.

**Table A2:** Detection limit and error for each element and the standard deviation (SD) of the results on the reference cement.

Parameter	Ca	Si	Al	Fe	SO_4_	Mg	Ti	K	Na	P	Mn
Detection limit (mg/L)	0.010	0.012	0.028	0.005	0.05	0.002	0.004	0.070	0.069	0.076	0.001
Error (%)	0.02	0.04	0.02	0.01	0.01	0.02	0.06	0.10	0.60	0.17	0.04
SD (mg/L)	0.39	0.21	0.13	0.10	0.02	0.06	0.01	0.04	0.01	0.01	0.00

## Appendix C

The analyses of the leached solutions were carried out using ICP–OES to determine the calcium concentrations and using HPIC to determine the sulfate concentrations in the leached solutions.

Regarding the calcium analysis, the concentrations of calcium in the leached solutions were very high, and the analytical method was adapted according to dilution factor, concentrations of standard solutions, wavelengths, and observation techniques to obtain the concentrations of calcium with the highest precision possible using ICP–OES. More specifically, (i) the calcium concentrations were situated in the optimal range of concentrations (between 0 and 100 mg/L) where the signal was optimized for the calcium element; (ii) the concentrations of standards were adapted to preserve the linearity of the calibration curve; and (iii) the wavelength of calcium spectrum was set to 317.933 nm.

For ICP–OES, the standard solutions, the concentrations of which are presented in Table A3, were prepared in ultrapure water with 2 vol.% of HNO_3_ plasma pure and a fondant at 4.8 g/L. The fondant was added to the standards to obtain the same matrices for the samples and the standards. Mono-element solution (with a concentration of 1000 mg/L) was used to prepare the standards for Si, Na, and K, and multi-element solution (with a concentration of 100 mg/L) was used to prepare the rest of the elements (Ca, Al, Fe, Mg, Ti and Mn).

**Table A3:** The concentrations of the standard solutions used for cement chemical analyses by ICP–OES

Standards	1	2	3	4	5	6	7	8	9	10	11	12
Multi (mg/L)	0.00	0.01	0.02	0.05	0.10	0.20	0.50	1.00	2.00	5.00	10.0	20.0
[Si] [Na] [K] (mg/L)	0.00	-	-	-	0.10	0.20	0.50	1.00	2.00	5.00	10.0	20.0

For HPIC analyses, the standard solutions, the concentrations of which are presented in Table A4, were prepared in ultrapure water with 0.08 vol.% of HCl plasma pure using the targeted concentrations presented in the table below. We have used a multi-element solution containing F^−^, SO_4_^2−^, NO_3_^−^, and PO_3_^4−^ to prepare the standards. However, the concentrations of the oxyanions were different ([F^−^] = 100 mg/L, [SO_4_^2−^] and [NO_3_^−^] = 500 mg/L, and [PO_3_^4−^] = 1000 mg/L); hence, the different concentrations for the standards.

**Table A4:** The concentrations of the standard solutions used for cement chemical analyses by HPIC.

Standards	1	2	3	4	5	6	7	8	9	10	11	12	13	14	15	16	17
[F^−^] (mg/L)	0.00	0.01	0.02	0.04	-	0.10	0.20	-	0.50	1.00	2.00	-	4.00	-	-	-	-
[SO_4_^2−^] [NO_3_^−^] (mg/L)	0.00	-	-	-	0.05	0.10	0.20	-	0.50	1.00	-	2.50	-	5.00	10.0	20.0	-
[PO_4_^3−^] (mg/L)	0.00	-	-	-	-	0.10	0.20	0.40	-	1.00	2.00	-	-	5.00	10.0	20.0	40.0

## References

[1] WHO/UNICEF (2019). Progress on Household Drinking Water, Sanitation and Hygiene 2000–2017.

[2] Kaempfer W., Berndt M. (1999). Estimation of service life of concrete pipes in sewer networks. Durab. Build. Mater. Compon..

[3] Parker C.D. (1947). Species of sulphur bacteria associated with the corrosion of concrete. Nature.

[4] Parker A.C.D. (1951). Mechanics of Corrosion of Concrete Sewers by Hydrogen Sulfide. Sewage Ind. Waste..

[5] Boon A.G. (1995). Septicity in sewers: Causes, consequences and containment. Water Sci. Technol..

[6] Islander B.R.L., Devinny J.S., Member A., Mansfeld F., Postyn A., Shih H. (1992). Microbial ecology of crown corrosion in sewers. J. Environ. Eng..

[7] Okabe S., Odagiri M., Ito T., Satoh H. (2007). Succession of sulfur-oxidizing bacteria in the microbial community on corroding concrete in sewer systems. Appl. Environ. Microbiol..

[8] Vincke E., Verstichel S., Monteny J., Verstraete W. (1999). A new test procedure for biogenic sulfuric acid corrosion of concrete. Biodegradation.

[9] Roberts D., Nica D., Zuo G., Davis J. (2002). Quantifying microbially induced deterioration of concrete: Initial studies. Int. Biodeterior. Biodegrad..

[10] Joseph A.P., Keller J., Bustamante H., Bond P.L. (2012). Surface neutralization and H2S oxidation at early stages of sewer corrosion: Influence of temperature, relative humidity and H2S concentration. Water Res..

[11] Rigdon J.H., Beardsley C.W. (1958). Corrosion of Concrete by Autotrophes. Natl. Assoc. Corros. Eng..

[12] Herisson J., Guéguen-Minerbe M., van Hullebusch E.D., Chaussadent T. (2014). Behaviour of different cementitious material formulations in sewer networks. Water Sci. Technol..

[13] Buvignier A., Patapy C., Lavigne M.P., Paul E., Bertron A. (2019). Resistance to biodeterioration of aluminium-rich binders in sewer network environment: Study of the possible bacteriostatic effect and role of phase reactivity. Cem. Concr. Res..

[14] Grandclerc A., Guéguen-Minerbe M., Nour I., Dangla P., Chaussadent T. (2017). Impact of cement composition on the adsorption of hydrogen sulphide and its subsequent oxidation onto cementitious material surfaces. Constr. Build. Mater..

[15] Grengg C., Mittermayr F., Baldermann A., Böttcher M.E., Leis A., Koraimann G., Grunert P., Dietzel M. (2015). Microbiologically induced concrete corrosion: A case study from a combined sewer network. Cem. Concr. Res..

[16] Jiang G., Keller J., Bond P.L. (2014). Determining the long-term effects of H2S concentration, relative humidity and air temperature on concrete sewer corrosion. Water Res..

[17] Lavigne M.P., Bertron A., Botanch C., Auer L., Hernandez-Raquet G., Cockx A., Foussard J.N., Escadeillas G., Paul E. (2016). Innovative approach to simulating the biodeterioration of industrial cementitious products in sewer environment. Part II: Validation on CAC and BFSC linings. Cem. Concr. Res..

[18] Goyns A.M., Alexander M. (2014). Performance of various concretes in the Virginia experimental sewer over 20 years. Calcium Aluminates: Proceedings of the International Conference 2014.

[19] Alexander M.G., Fourie C. (2011). Performance of sewer pipe concrete mixtures with portland and calcium aluminate cements subject to mineral and biogenic acid attack. Mater. Struct. Constr..

[20] Monteny J., Vincke E., Beeldens A., De Belie N., Taerwe L., Van Gemert D., Verstraete W. (2000). Chemical, microbiological, and in situ test methods for biogenic sulfuric acid corrosion of concrete. Cem. Concr. Res..

[21] Aboulela A., Peyre-Lavigne M., Patapy C., Bertron A. (2018). Evaluation of the resistance of CAC and BFSC mortars to biodegradation: Laboratory test approach. MATEC Web of Conferences.

[22] Grengg C., Mittermayr F., Koraimann G., Konrad F., Szabó M., Demeny A., Dietzel M. (2017). The decisive role of acidophilic bacteria in concrete sewer networks: A new model for fast progressing microbial concrete corrosion. Cem. Concr. Res..

[23] Aboulela A., Peyre-Lavigne M., Bertron A., Bertron A., Jonkers H., RILEM (2018). Investigation of test methods to qualify cementitious materials. Proceedings of the RILEM TC 253-MCI Conference Microorganisms-Cementitious Materials Interactions PRO123.

[24] Sand W., Bock E. (1984). Concrete corrosion in the hamburg sewer system. Environ. Technol. Lett..

[25] Ehrich S., Helard L., Letourneux R., Willocq J., Bock E. (1999). Biogenic and Chemical Sulfuric Acid Corrosion of Mortars. J. Mater. Civ. Eng..

[26] Lavigne M.P., Bertron A., Auer L., Hernandez-Raquet G., Foussard J.N., Escadeillas G., Cockx A., Paul E. (2015). An innovative approach to reproduce the biodeterioration of industrial cementitious products in a sewer environment. Part I: Test design. Cem. Concr. Res..

[27] NF EN 196-2. Methods of Testing Cement—Part 2: Chemical Analysis of Cement. https://www.boutique.afnor.org/standard/nf-en-196-2/methods-of-testing-cement-part-2-chemical-analysis-of-cement/article/809132/fa178507.

[28] Kiliswa M.W., Scrivener K.L., Alexander M.G. (2019). The corrosion rate and microstructure of Portland cement and calcium aluminate cement-based concrete mixtures in outfall sewers: A comparative study. Cem. Concr Res..

[29] Kiliswa M.W. (2016). Composition and Microstructure of Concrete Mixtures Subjected to Biogenic Acid Corrosion and Their Role in Corrosion Prediction of Concrete Outfall Sewers. Ph.D. Thesis.

[30] Letourneux R., Scrivener K., Dhir R.K., Dyer T.D. (1999). The Resistance of Calcium Aluminate Cements To Acid Corrosion in Wastewater Applications. Modern Concrete Materials: Binders, Additions and Admixtures.

[31] Saucier F., Lamberet S. Calcium Aluminate Concrete for sewers: Going from qualitative to quantitative evidence of performance. Proceedings of the Concrete in Aggressive Aqueous Environments, Performance, Testing and Modeling.

[32] Herisson J., Guéguen-Minerbe M., van Hullebusch E.D., Chaussadent T. (2017). Influence of the binder on the behaviour of mortars exposed to H2S in sewer networks: A long-term durability study. Mater. Struct..

[33] Scrivener K.L., Cabiron J.-L., Letourneux R. (1999). High-performance concretes from calcium aluminate cements. Cem. Concr. Res..

[34] Jahani B.F., Devinny J., Mansfeld F., Rosen I.G., Sun Z., Wang C. (2001). Investigations of Sulfuric acid Corrosion of Concrete. J. Environ. Eng..

[35] (2016). NF EN 196-1. Methods of Testing Cement—Part 1: Determination of Strength. https://www.boutique.afnor.org/standard/nf-en-196-1/methods-of-testing-cement-part-1-determination-of-strength/article/866862/fa184622.

[36] Satoh H., Odagiri M., Ito T., Okabe S. (2009). Microbial community structures and in situ sulfate-reducing and sulfur-oxidizing activities in biofilms developed on mortar specimens in a corroded sewer system. Water Res..

[37] Vincke E., Boon N., Verstraete W. (2001). Analysis of the microbial communities on corroded concrete sewer pipes—A case study. Appl. Microbiol. Biotechnol..

[38] Davis J.L., Nica D., Shields K., Roberts D.J. (1998). Analysis of concrete from corroded sewer pipe. Int. Biodeterior. Biodegrad..

[39] Thompson M., Walsh J.N. (1989). Inductively Coupled Plasma Spectrometry.

[40] Vandecasteele C., Block C.B. (1997). Modern Methods for Trace Element Determination.

[41] Tyler G. (2001). ICP-OES, ICP-MS and AAS Techniques Compared. ICP Opt. Emiss. Spectrosc. Tech. Note.

[42] Jiang G., Zhou M., Chiu T.H., Sun X., Keller J., Bond P.L. (2016). Wastewater-Enhanced Microbial Corrosion of Concrete Sewers. Environ. Sci. Technol..

[43] Ling A.L., Robertson C.E., Harris J.K., Frank D.N., Kotter C.V., Stevens M.J., Pace N.R., Hernandez M.T. (2015). High-Resolution Microbial Community Succession of Microbially Induced Concrete Corrosion in Working Sanitary Manholes. Mormile MR, editor. PLoS ONE.

[44] Milde K., Sand W., Wolff W., Bock E. (1983). Thiobacilli of the corroded concrete walls of the Hamburg sewer system. J. Gen. Microbiol..

[45] Grandclerc A., Guéguen-Minerbe M., Chaussadent T., Bertron A., Jonkers H., RILEM (2018). Accelerated Biodeterioration Test Of Cementitious Materials In Sewer Networks. Proceedings of the RILEM TC 253-MCI Conference Microorganisms-Cementitious Materials Interactions PRO123.

[46] Herisson J., van Hullebusch E.D., Moletta-Denat M., Taquet P., Chaussadent T. (2013). Toward an accelerated biodeterioration test to understand the behavior of Portland and calcium aluminate cementitious materials in sewer networks. Int. Biodeterior. Biodegrad..

[47] Kiliswa M.W., Alexander M.G. Biogenic Corrosion of Concrete Sewer Pipes: A Review of the Performance of Cementitious Materials. Proceedings of the XIII Conference Durab Build Mater Components.

[48] Grengg C., Ukrainczyk N., Koraimann G., Mueller B., Dietzel M., Mittermayr F. (2020). Long-term in situ performance of geopolymer, calcium aluminate and Portland cement-based materials exposed to microbially induced acid corrosion. Cem. Concr. Res..

[49] Gosselin C. (2009). Microstructural Development of Calcium Aluminate Cement Based Systems with and without Supplementary Cementitious Materials.

[50] Wells T., Melchers R. Concrete Sewer Pipe Corrosion—Findings From an Australia Field Study. Proceedings of the Ozwater 2016.

[51] Taylor H.F.W. (1997). Cement Chemistry.

[52] Erbektas A.R., Isgor O.B., Weiss W.J. (2019). An accelerated testing protocol for assessing microbially induced concrete deterioration during the bacterial attachment phase. Cem. Concr. Compos..

[53] Allahverdi A., Skvára F. (2000). Acidic corrosion of hydrated cement based materials—Part 1. Ceram. Silikáty.

[54] Lamberet S., Guinot D., Lempreur E., Talley J., Alt C., Fentiman C.H., Managbhai R.J., Scrivener K.L. (2008). Field Investigations of High Performance Calcium Aluminate Mortar for Wastewater Applications. Calcium Aluminates: Proceedings of the International Conference 2008.

[55] Grandclerc A., Dangla P., Gueguen-Minerbe M., Chaussadent T. (2018). Modelling of the sulfuric acid attack on different types of cementitious materials. Cem. Concr. Res..

[56] Lavigne M.P., Bertron A., Patapy C., Lefebvre X., Paul E. (2015). Accelerated test design for biodeterioration of cementitious materials and products in sewer environments. Matériaux Tech..

[57] Bureau of Analysed Samples Ltd. (2012). Certificat of Analysis: BCS-CRM No. 353. Middlesbrough.

